# Use of Non-Prescription Remedies by Ghanaian Human Immunodeficiency Virus-Positive Persons on Antiretroviral Therapy

**DOI:** 10.3389/fpubh.2017.00115

**Published:** 2017-05-26

**Authors:** Amos K. Laar, Awewura Kwara, Priscillia A. Nortey, Augustine K. Ankomah, Michael P. K. Okyerefo, Margaret Y. Lartey

**Affiliations:** ^1^Department of Population, Family, and Reproductive Health, School of Public Health, University of Ghana, Accra, Ghana; ^2^Department of Medicine, Warren Alpert Medical School of Brown University, Providence, RI, United States; ^3^Department of Epidemiology and Disease Control, School of Public Health, University of Ghana, Accra, Ghana; ^4^Department of Sociology, University of Ghana, Accra, Ghana; ^5^Department of Medicine, University of Ghana School of Medicine and Dentistry, University of Ghana, Accra, Ghana

**Keywords:** human immunodeficiency virus, antiretroviral therapy, non-prescription remedies, polypharmacy, polyherbacy, self-medication practices, Ghana

## Abstract

**Background:**

Inappropriate use of non-prescription remedies by persons living with human immunodeficiency virus (PLHIV) may result in adverse events or potentiate non-adherence to prescribed medications. This study investigated the use of non-prescription remedies among PLHIV receiving antiretroviral therapy (ART) from four treatment centers in southern Ghana.

**Methods:**

A mixed method design using quantitative and qualitative methods was used. This article focuses on the quantitative survey of 540 respondents. Univariate analysis was used to generate descriptive tabulations of key variables. Bivariate analysis and logistic regression modeling, respectively, produced unadjusted and adjusted associations between background attributes of PLHIV and the use of non-prescription remedies. A *p*-value of < 0.05 was considered statistically significant. All analyses were performed using IBM SPSS Statistics for Windows, Version 20.0.

**Results:**

One out of three respondents reported the use of non-prescription remedies at least once within 3 months of the survey. Most of these were locally made and included “Angel natural bitters, concoctions from the Christian prayer centers, garlic, and mahogany syrups.” These remedies were used concomitantly with antiretroviral medications (ARVs)—46% or administered with ARVs but at different times during the day (43%). Some of the remedies were reportedly prescribed by health workers, or self-initiated during periods of ARVs shortage. Others took them based on their perception of their efficacy. Bivariate level analysis identified ART clinic site, place of residence, and ARV adherence monitoring to be significantly associated with the use of non-prescription remedies (*p* < 0.05). Multiple logistic regression analysis controlling for covariates confirmed the location of ART clinic as the only predictor of the use of non-prescription remedies. Compared to clients at the large urban teaching hospital (Korle-Bu Fevers Unit ART center), those at the district level (Atua ART center) were ninefold more likely to use non-prescription remedies [adjusted odds ratio (AOR) = 8.84; 95% confidence interval (CI) 2.83–33.72]. Those from a district level mission hospital (St. Martin’s ART center) were threefold as likely to use these remedies (AOR = 2.610; 95% CI 1.074–9.120).

**Conclusion:**

The use of non-prescription remedies by PLHIV on ART is common in southern Ghana. Usage is mostly self-initiated because of perceived efficacy of remedy, and was more common among clients attending rural ART clinics.

## Background

Identified over 30 years ago, the human immunodeficiency virus (HIV) infection remains a serious health problem globally with an average of 34.0 million persons living with HIV (PLHIV) at the end of 2012 (1). Sub-Saharan Africa is the most severely affected region with nearly one in every 20 adults (4.9%) living with HIV and accounting for 69% of the PLHIV worldwide (1). The Ghana National HIV/STI Control Program (NACP) estimates the total number of PLHIV and AIDS in Ghana as at 2013 to be 220,000. An estimated 10,000 people died from the epidemic that same year (2). The annual HIV sentinel survey reports suggest a downward trend in HIV prevalence: from a 3.6% in 2003, it saw a marginal reduction to 3.2% in 2006, a further reduction to 2.2% in 2008. The prevalence in years 2010 and 2011 was 2.0%; this has since reduced to 1.3% in 2013 (2). Data on incidence are not available in Ghana.

Although HIV is still a major public health problem, the advent of antiretroviral therapy (ART) has made it a manageable chronic disease (3). Ghana started ART with treatment initiation threshold of CD4 of less than 250 from 2003. This was revised to CD4 of less than 350 in 2008. The number of patients receiving ART overwhelmingly increased 200-fold from 197 patients in 2003 to over 45,000 in 2010 (4). Local programmatic data revealed national coverage of ART to be 70.5% in 2015; adult ART coverage was 72.3% with an unmet need of about 30% for the whole HIV-positive adult population; pediatric coverage was 35.4% with an unmet need for all HIV-positive children of about 65% (2).

The primary expected outcome of ART is suppression of viral load, restoration of immunological functions, and ultimately improvement in quality of life (5). Several studies show that near-perfect levels of HIV medication adherence are necessary for a durable suppression of viral replication (6–12) and hence treatment success (13, 14). Confounded by the fact that drug use in HIV is complex and may involve multiple therapeutic and non-therapeutic agents including prescription, non-prescription, and complementary and alternative medicine (CAM) (15–19), such levels of adherence remains a dream in many parts of the world (15, 20–23). In particular, widespread concomitant use of herbal medicines by ART patients appear to impact negatively on adherence (24, 25).

Other challenges that have the potential to affect retention in ART clinics and medication adherence have been characterized by Musheke and Merten (26). The reasons attributed to low retention in ART care or clinics, for example, include financial cost, fear of stigmatization, the need to travel long distances to receive medication, food insecurity, lack of access to correct information, pill burden, and preference for faith and religious remedies compared to orthodox treatment (4, 26, 27). In a descriptive cross-sectional study conducted in the Upper West Region of Ghana, patients’ suboptimal adherence to ART therapy was mainly attributed to forgetfulness and shortage of drugs (4). Only 17% of the patients missed taking their antiretroviral medications (ARVs) due to side effects (4). In Guinea-Bissau, Rasmussen et al. identified HIV-related knowledge as the most important factor influencing adherence to ART (27).

Currently, self-management of HIV involves the conventional prescription use of ARV plus non-prescription alternatives such as over-the-counter (OTC), CAMs, and herbal/traditional medicine (28–30). Access to OTC has become very easy for HIV patients due to the ubiquity and affordability of various health outlets in both cities and villages of Africa. Another non-prescription remedy usually patronized in Africa is faith-based remedies (26). In Ghana and other African countries, these are not carefully documented but no doubt exists and may negatively impact on ART adherence. A case in Zambia revealed that HIV-seropositive patients seek help and treatment from professing men of God and pastors with healing abilities, thus practicing “self care.” They attend private prayers and group prayers all in an effort to receive healing from the disease. They are convinced that their sins have brought such repercussions and as such being found always in the house of God might bring forgiveness and a miraculous healing from the HIV disease (26).

In developing countries, usage of herbal traditional medicine is very prevalent (31, 32), and it is the first source of medication by most of its inhabitants (28, 33). Alternative complementary therapies including the use of herbs, vitamins, mineral supplements, and a host of non-prescription drugs have long been used by PLHIV in hopes of helping manage side effects of other therapies and/or improve overall general health (17, 31, 32, 34). Studies suggest that upwards of 70% of PLHIV use some form of complementary therapy (31, 35, 36). The vast majority of the African people (80%), according to the World Health Organization, rely on traditional medicine for their primary health care needs (37). In most settings in the African region, usage of herbal medicine is a routine practice irrespective of whether or not one is ill. It is taken for the general belief that it improves health and drives away evil spirits (26–28, 30). A study in Lusaka, Zambia, revealed that even health workers with HIV and those working in ART centers do resort to faith healing and usage of herbal remedies (26).

Of note, several studies show that usage of the remedies being described is more for a complementary kind rather than an alternative to ART even though few use it exclusively for treatment (28, 30–34). Estimates from studies also show that at least 40–80% of patients on ART will use any form of traditional CAM (29, 30, 38) The growing liking and advocacy for these forms of alternative treatment in PLHIV in Africa is in part due to the flexibility that characterizes usage of the remedies in comparison to the ART which requires a rigid routine. In addition, HIV-positive patients tend to opt for herbal medicine to alleviate side effects such as nausea and diarrhea they experience due to the usage of ARV drugs. Easy access to herbal medicine, as some patients grow the herbs in their homes, is also a factor in the prevalence of the use of herbal medicine (26, 30).

Some clinical studies have shown that herbal medicines might have the potential to alleviate symptoms, reduce viral load, and increase CD4+ cells for HIV-infected individuals and AIDS patients (33, 38). On the other hand, same studies warn of liver toxicity and other adverse events from some herbal products as well as possible herb–drug interactions (33, 38). The study by Liu et al. (38) revealed that about 20% of HIV patients used herbs that were very likely to compromise the effectiveness of ART. Aside from the adverse drug events such as psychiatric and neurological effects that may occur with concomitant use of herbal medicine as a remedy in HIV-positive persons on ART, the worrying part is the irregular dosaging, lack of knowledge on its efficacy, intrinsic toxicity of ingredients, and the manner and condition under which preparation of the traditional medicine is performed (29). Dosaging, efficacy, and intrinsic toxicity of ingredients are difficult to ascertain because the preparation process and ingredients added to herbal mixtures in typical African villages and communities are not well documented, it is by word of mouth, passed on from generation to generation (30).

Like other countries in Africa, the use of alternative complementary medication in Ghana is popular. The Ghana Herbal Pharmacopoea (GHP) reveals that about 70% of Ghanaians depend on alternative health care practices for their primary health care needs (39). Other local data show that such practices do have religious and historical antecedents. Awusabo-Asare and Anarfi have indicated that diseases whose etiology could not be readily explained are given supernatural explanations among the various ethnic groups in Ghana (40). With no known cure, HIV/AIDS is one of those diseases that community members associate with such supernatural remedies as faith healing, or local alternative complementary medicine. Without a doubt, such an explanation of disease causation influences people’s attitude to the disease and to infected persons, which in turn influences the health-seeking behavior of infected persons. As such, some HIV-infected persons in Ghana use multiple health care outlets, either serially or simultaneously, hoping that one of them might provide a cure or relief as well as explain the source of the infection (40). Elsewhere, numerous medicinal plants used by non-allopathic medical practitioners for the treatment of HIV have been identified (41–44). Others have reported concomitant use of these alternative complementary therapies in patients receiving ART (24, 25).

Various motivators for the practice exist. Previously identified motivating variables include flexibility in the use of alternative remedies, the pill burden of orthodox HIV medication among others. In the Ghanaian context, there is no systematic documentation of the issues. However, anecdotal evidence links the practice to lack of robust guidelines on medication use, periodic shortages of ARVs, side effects associated with ARVs, and perceived efficacy of alternative medications. Even though proponents of alternative remedies argue that this empowers patients to treat themselves, when used inappropriately, the medications could pose significant risks. A number of studies have associated the use of multiple medications with adverse consequences including treatment non-adherence (7, 45, 46). Additional motivators of the practices have been identified (17, 47, 48).

The concomitant use of prescribed and alternative remedies and its motivators remain an uncharacterized phenomenon in Ghana. Information on the use of medication in Ghana is derived primarily from secondary sources and in some instances anecdotal evidence. This limits their use in predicting trends and behavior motivators. Against this background, a systematic characterization of the use of non-prescription remedies or abuse among HIV-positive persons on ART in Ghana is relevant.

## Materials and Methods

### Design and Study Sites

The study was descriptive and cross-sectional in design and used both qualitative and quantitative methods of data collection. This article uses data from the quantitative surveys. It was conducted at four health facilities in southern Ghana where ART is offered to HIV-positive clients. These health facilities are the Fevers Unit of the Korle Bu Teaching Hospital (located in the capital city of Ghana—Accra), and the Tema General Hospital (located in Ghana’s industrial city of Tema). The Atua Government Hospital (located in Atua), and St. Martin’s de Porres Hospital (located in Agormenyah) were the other two sites in the Eastern Region of Ghana. Those two hospitals from the Eastern Region were the first sites to start the ART program using the public health approach. The Korle Bu Teaching Hospital is one of the tertiary hospitals in southern part of Ghana. The Fevers unit affiliated to the Department of Medicine has the largest population of PLHIV at a single site. At the time of the study, the Fevers Unit of the Korle Bu Teaching Hospital had about 6,000 patients on ART. Tema General Hospital (a regional hospital) had about 1,500 HIV-positive clients who were enrolled on ART. The Atua Government hospital and the St. Martin’s hospital had about 4,800 and 4,000 HIV-positive clients on ART, respectively.

### Sample Size Estimation, Sampling, and Summary of Other Field Procedures

The sample size for the quantitative component of the study was determined using Statcalc in Epi Info 2,000 package (49). The level of concurrent use of non-prescription drugs is unknown in this population, but was assumed to be 50%. We further estimated the worst acceptable level of use to be as low as 45% or as high as 55%. With an alpha of 0.05 and a statistical power of 80%, 384 clients were computed as the minimum sample needed to assess the extent and patterns of the use of non-prescription remedies among HIV-positive persons receiving ART from the four treatment centers. This sample size was further increased by 20% to account for contingencies such as non-responses or recording errors. The minimum sample size of 461 was then rounded up to 500 clients.

Prior to sampling, the probability proportional to size weighting procedure was employed in the allotment of the PLHIV to the four study sites. Thus, study sites with larger number of PLHIV had more PLHIV participating in the study. Using average monthly attendance, the required numbers of PLHIV to be interviewed by study site were 140 each for the Fevers Unit, Korle Bu Teaching Hospital, St. Martin’s Martins de Porres Hospital, and the Atua Government Hospital, and 80 for the Tema General Hospital. Actual sample size at the end of data collection was, respectively, 153, 148, 146, and 93. Study participants were selected using a systematic random sampling with random start. To do this, the master list of ART clients at each facility served as the site-specific sampling frame. The sampling interval (*n*) for each site was derived by dividing the total number of participants on the monthly register by the required sample at each site. The interviews were performed between May 5, 2014, and June 30, 2014, by eight trained research assistants (RAs). RAs were recruited based on survey experience and knowledge of the local area. Training entailed introduction to the study objectives, goals, methods, and expected outcomes. There were extensive role-plays to ensure accuracy during field work. Study tools were pre-tested after the training and the needed validations performed prior to the actual data collection.

With the help of health personnel managing the ART clinics, eligible study participants were identified and interviewed. Given the sensitive nature of HIV, clinicians (mainly nurse prescribers) who had good rapport with the PLHIV facilitated the researchers’ interaction with the participants. The nurses, who had prior briefing about the study, identified prospective study participants and referred them to the RAs. All interviews were performed after obtaining informed consent. The interview elicited information on the various medications and remedies used by the ART clients (ART and other approved allopathic and traditional/herbal medications, non-prescription drugs, nutrient supplements, etc.). Clients’ hospital records were also reviewed and relevant data extracted. The interviews further assessed motivations for the various practices. *A priori* identified potential variables which formed part of the interview guide included the lack of robust guidelines on medication use, periodic shortages of ARVs, cost of ARVs, and side effects associated with ARVs, and perceived the efficacy of alternative medications. Data collected also recorded the formulations of the remedies and how the medications were being administered by clients—whether or not they were administered concomitantly with ART, substituted for ART, or administered independently.

### Ethical Considerations

Participation in the study conformed to the required ethical guidelines for use of human subjects. The study proposal was reviewed and approved by the Ethical Review Committee of the Ghana Health Service, Research and Development Division, Accra (Protocol ID NO: GHS-ERC 03/11/13). Permission was granted from the facilities within which the study was conducted. Informed consent was obtained from all participants after the objectives and the methodology of the study was explained to them. Participation in the study was completely voluntary, no financial or material benefits were given. The privacy and confidentiality of every participant were ensured throughout the study period. Identification numbers (and not names) were used to disguise identity. Every member of the data collection and analysis team was cautioned during the training sessions to maintain strict confidentiality and anonymity of study data and participants. The participants, were all adults (18 years or older).

### Data Management and Analysis

Interviews were performed using paper-based questionnaires. Data were reviewed daily for inconsistencies or omissions and problems with data completion addressed. Data collectors reviewed all completed data collection forms and corrected any errors or inconsistencies before hand-delivering them, along with their accompanying consent forms to field supervisors. Supervisors also reviewed the forms for accuracy, consistency, and completion. Once the data collection forms were considered complete, they were securely delivered to the principal investigator’s office where they were kept in locked filing cabinets. All quantitative survey data were doubly entered into pre-programmed data screens designed in CSPro (Census and Survey Processing System). Data were exported to IBM SPSS Statistics, version 20, for consistency checks and validation. Cleaned and validated data sets were analyzed using the same program.

We used univariate analysis to generate descriptive tabulations for key variables. Bivariate analysis and logistic regression modeling, respectively, produced unadjusted and adjusted associations between background attributes of PLHIV and their use of non-prescription remedies. We employed a standard logistic regression modeling in SPSS (the “Enter” method) in our analysis. With this method, all the variables previously reported to be associated with the outcome variable or found to be associated with the outcome during the bivariate analysis were entered and a full model generated in a single step. The attributes of the model are included in the tables presented. A *P*-value of <0.05 was used to denote statistical significance.

## Results

Table 1 presents the background, socio-demographic, and selected clinical characteristic of study participants. A little over half of the study sample (54%) was from the Eastern Region study sites. The remaining were from the Greater Accra study sites. Almost three-quarters (74%) of the respondents were female. The sampled population was overwhelmingly Christian (90%). About 80% of them had some form of formal education, and 92% were in the age band of 25–60 years; 23.3% representing those aged 25–35 years and 69.3% representing those aged 36–60 years. Of 400 women who formed part of the study sample, data on physiologic state was available on 357. Of these, 3% were pregnant and 4% nursing young children. Close to 50% of the study participants were either anemic or had CD4 count of less than 350 cells/mm^3^, as many of the participants were underweight as they were obese (Table 1).

**Table 1:** **Background, socio-demographic, and selected clinical characteristics of study participants (n = 540 unless indicated otherwise)**.

	Frequency	Percent
**Study site**		
Atua Government Hospital	146	27.0
St Martin’s Martins de Porres Hospital	148	27.4
Tema General Hospital	93	17.2
Fevers Unit, Korle Bu Teaching Hospital	153	28.3
**Place of residence**		
Urban	275	50.9
Rural	265	49.1
**Sex of respondent**		
Male	140	25.9
Female	400	74.1
**Religious affiliation of respondent**		
Not religious	10	1.9
Christian	485	89.8
Muslim	43	7.9
Traditionalist	2	0.4
**Respondent’s level of education**		
No. of formal education	109	20.1
Primary	123	22.9
JHS	170	31.4
SHS/vocational	102	18.9
Post-secondary/tertiary	36	6.7
**Total**	**509**	**100.0**
**Age^a^**		
18–19 years	5	0.9
20–24 years	12	2.2
25–35 years	126	23.3
36–60 years	374	69.3
61 years or older	23	4.3
**Physiologic status of female respondents**		
Pregnant	10	2.8
Lactating	14	3.9
Not pregnant	333	93.3
**Total**	**357**	**100.0**
**Body mass index (BMI)^b^**		
Underweight, BMI <18.5	53	12.2
Normal weight, BMI >18.5/<25	236	54.1
Overweight, BMI >25/<30	99	22.7
Class I obesity, BMI >30/<35	29	6.7
Class II obesity, BMI >35/<40	11	2.5
Class III obesity, BMI >40	8	1.8
**Total**	**436**	**100.0**
**CD4+ cell count^c^**		
CD4+ cell count <350	235	47.3
CD4+ cell count >350	262	52.7
**Total**	**497**	**100.0**
**Hemoglobin concentration (g/dl)^d^**		
Anemic (Hb <11.0 g/dl)	239	46.4
Normal hemoglobin levels	276	53.6
**Total**	515	100.0

*^a^Mean age is 42.3, and ranged from 18 to >60 years*.

*^b^Mean BMI is 23.93, and ranged from 13.61 to 50.00*.

*^c^Median CD4 cell count is 373, and ranged from 2 to 1,663; 43 cases are missing CD4 measurements*.

*^d^Mean Hb is 11.1, and ranged from 5.8 to 19.2; 25 cases are missing Hb measurements*.

**Figure 1:**
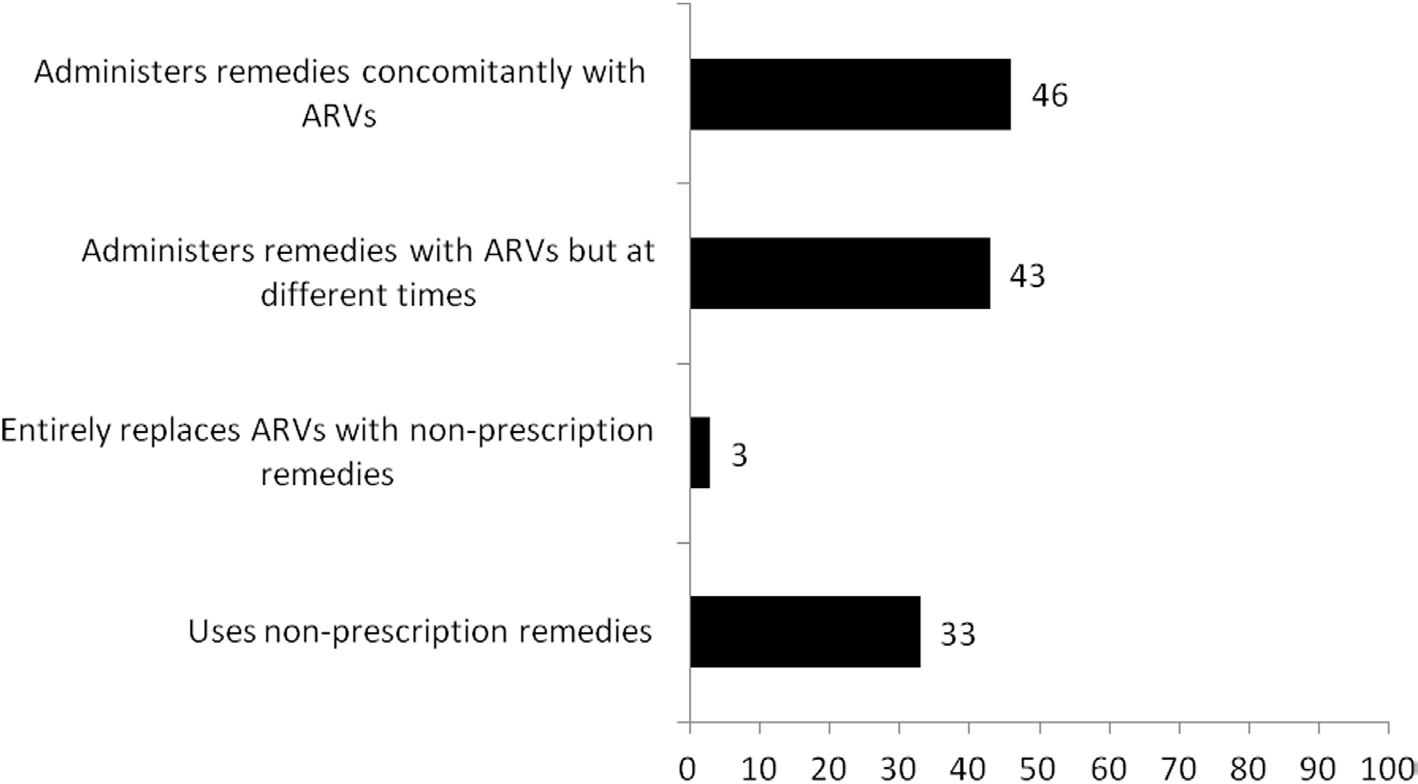
**Use of non-prescription remedies and self-reported adherence to antiretroviral medication regimen**.

We summarize in Figure 1 the use of non-prescription remedies and self-reported adherence to ARV regimen by the study participants. One out of three reportedly used some form of non-prescription remedies 3 months preceding the survey. Most of the non-prescription remedies were locally made and included “Angel natural bitters, Angel natural capsules, concoctions from the Christian prayer centers, garlic, lime, and mahogany syrups; others were Losartan, Bendro, and odoba eloquin.” These were mostly used concomitantly with ARVs (46%) or administered with ARVs but at different times in a day (43%). Some of the remedies were reportedly prescribed by health workers (nurse prescribers), or self-initiated informed by shortage or ARVs, or based on their perceived efficacy.

Almost all the respondents believed they adhere to their ARV regimen. Whenever, they accessed ARVs, they reportedly took them as prescribed and with food. However, when probing was performed to obtain data on the number of times they missed a dose of their ARV medication the month preceding the interview, 88% indicated missing none, 5% missed only once, and 7% missed two or more times (data not shown in Figure 1). Fifty-five percent of the clients have adherence monitors (most of whom were family members, friends, or pastors). Visitation of facilities (other than ART clinics) for care was popular. “Clinics” (not ART centers) were mentioned 473 times as point of care 3 months preceding the interview. Pharmacies or chemical shops were mentioned 260 times, indigenous healers 129 times, and healing churches 142 times. Motivators for using these other facilities included “convenience in terms of distance, immediate care, financial challenges, shortage of ARV medications, and trust of alternative medical practitioners.”

Table 2 presents predicators of the use of non-prescription remedies by PLHIV on ART. Outputs from two analytic procedures (bivariate Chi-square analysis and multiple logistic regression modeling) are presented together with their unadjusted and adjusted measures of association. The bivariate level analysis shows that ART clinic location, place of residence, ARV adherence monitoring, and anemia are significantly associated with the use of non-prescription remedies (*p* < 0.05) in each case. Sex of ART client, religious affiliation, level of education, body mass index, and CD4 + cell count were all not significantly associated with the use of non-prescription remedies (*p* > 0.05) in each case (Table 2).

**Table 2:** **Background and socio-demographic predictors of non-prescription drug use**.

Attribute	Use non-prescription remedies	Does not use non-prescription remedies	*p*-Value	Adjusted odds ratio (AOR)	95% confidence interval	
**ART site**			<0.001		Lower limit	Upper limit
Atua ART Clinic	3 2.1	143 97.9		8.84	2.83	33.72
St Martins de Porres Hospital	69 50.0	69 50.0		2.61	1.07	9.12
Tema General Hospital	76 84.4	14 15.6		0.02	0.01	0.06
Korle Bu Fevers Unit	26 17.2	125 82.8		Ref	Ref	Ref

**Sex of respondent**			0.431			
Male	42 30.4	96 69.6		1.77	0.29	10.93
Female	132 34.1	255 65.9		Ref	Ref	Ref

**Religious affiliation of respondent**			0.113			
Traditionalist/no religion	5 45.5	6 54.5		0.10	0.01	1.53
Christian	16 134.0	312 66.0		0.42	0.09	1.86
Muslim	8 19.5	33 80.5		Ref	Ref	Ref

**Place of residence**			0.033			
Urban	101 37.4	169 62.6		0.71	0.22	2.27
Rural	73 28.6	182 71.4		Ref	Ref	Ref

**Respondent’s level of education**			0.172			
No. of formal education	24 23.1	80 76.9		0.87	0.21	3.63
Primary	44 37.9	72 62.1		1.12	0.30	4.21
JHS	57 34.8	107 65.2		1.54	0.40	5.89
SHS/vocational	35 34.7	66 65.3		0.77	0.17	3.49
Post-secondary/tertiary	13 36.1	23 63.9		Ref	Ref	Ref

**Does respondent have to adherence monitor**			<0.001			
Yes	126 45.0	154 55.0		0.83	0.35	1.98
No	48 19.7	196 80.3		Ref	Ref	Ref

**Physiologic state of female respondent**						
Pregnant	3 30.0	7 70.0		1.37	0.23	8.38
Lactating	3 23.1	10 76.9		0.40	0.01	12.13
Not pregnant	118 35.6	213 64.4		Ref	Ref	Ref

**Age categories**			0.364			
18–19 years	0 0.0	5 100.0		–	–	–
20–24 years	5 41.7	7 58.3		0.79	0.05	12.02
25–35 years	39 33.9	76 66.1		1.07	0.11	10.59
36–60 years	125 33.8	245 66.2		0.71	0.08	6.21
61 years or older	5 21.7	18 78.3		Ref	Ref	Ref

**BMI**			0.892			
Underweight	13 (24.5)	40 (75.5)		–	–	–
Normal weight	61 (26.0)	174 (74.0)		2.23	0.09	55.65
Overweight	28 (28.3)	71 (71.7)		1.06	0.06	19.60
Class I obesity	9 (32.1)	19 (67.9)		0.93	0.05	17.49
Class II obesity	4 (36.4)	7 (63.6)		0.67	0.03	14.68
Class III obesity	3 (37.5)	5 (62.5)		0.34	0.02	8.01

**CD4+ cell count**			0.230	Ref	Ref	Ref
CD4+ cell count <350	72 (30.9)	161 (69.1)		0.85	0.39	1.88
CD4+ cell count >350	94 (36.0)	167 (64.0)		Ref	Ref	Ref

**Anemia status**			<0.001			
Anemic	99 (41.6)	139 (58.4)		0.55	0.25	1.21
Normal	71 (25.9)	203 (74.1)	Ref	Ref	Ref	Ref

*p-value significant at 0.05 level (and from Chi-square test)*.

*AOR, adjust odds ratios with accompanying 95% confidence intervals were determined using multiple logistic regression (all variables in Table 2 were included in the model)*.

*Model Summary Cox and Snell R Square (0.420), Nagelkerke R Square (0.578)*.

*Ref means reference category*.

The multiple logistic regression model examined predictors of same outcome (use of non-prescription remedies) after adjusting for a number of covariates. This analysis confirmed the location of ART clinic as the only predictor of the use of non-prescription remedies. Compared to clients from the Korle-Bu Fevers Unit, those from the Atua ART site were ninefold as likely to use non-prescription remedies [adjusted odds ratio (AOR) = 8.84; 95% confidence interval (CI) 2.829–33.720]. Those from the St. Martin’s site were threefold as likely to use these remedies (AOR = 2.610; 95% CI 1.074–9.120). Accessing ART from the Tema site was found to be protective against the use of non-prescription remedies (AOR = 0.019; 95% CI 0.007–0.055; Table 2).

## Discussion

This study aimed to investigate the use of non-prescription remedies among HIV-positive persons receiving ART from selected treatment centers in southern Ghana. The key findings include a pronounced use of non-prescription remedies among them; most of the non-prescription remedies (which were locally made) were mostly used concomitantly with ARVs (46%) or administered complementarily with ARVs (43%). Some of the remedies were reportedly prescribed by nurse prescribers, or self-initiated informed by shortage or ARVs, or based on their perceived efficacy. Location of an ART clinic significantly predicts the use of these remedies. These key findings are discussed.

The popularity of non-prescription remedies among the studied population resonates with previous studies. For example, in Ghana, the GHP show that upwards of 70% of Ghanaians depend on alternative health care practices for their primary health care needs (39). Awusabo-Asare and Anarfi had earlier linked such practices to religious and historical antecedents (40). Elsewhere, numerous medicinal plants have been identified and used by non-allopathic medical practitioners for the treatment of HIV (41–44). Others have reported concomitant use of these alternative complementary therapies in patients receiving ART (24, 25).

Other studies report on the patronage of faith-based remedies by PLHIV. The unity of spiritual and natural causes of illness, remedies for care and treatment (50, 51) have historically been emphasized by traditional and religious leaders and healers in Ghana and other West African countries. With the recent rapid growth of Pentecostal–Charismatic churches in Ghana, faith healing has edged out traditional religious healing (52). Of note, the religious and culturally driven justifications for the use of alternative remedies among HIV-positive persons as well as HIV-uninfected persons are not new in Ghana, although these are not carefully documented. Their existence may, however, negatively impact on ART adherence. A case in Zambia revealed that HIV-seropositive patients seek help and treatment from professing men of God and pastors with healing abilities, thus practicing “self-care.” They attend private prayers and group prayers all in an effort to receive healing from the disease. They are convinced that their sins have brought such repercussions on them and as such being found always in the house of God might bring forgiveness and a miraculous healing from the HIV disease (26).

The finding that non-prescription medications were used concomitantly with ARVs and not as substitutes or replacement reaffirms previous investigations. A number of studies have shown that the use of non-prescription remedies is more for a complementary kind rather than an alternative to ART even though few use it exclusively for treatment. For instance, estimates from earlier studies show that at least 40–80% of patients on ART will use any form of traditional CAM not exclusively, but as an add on to standard drug regimen (29, 30, 33). These studies have attempted to discover motivations for such practices. Popular motivators such as the flexibility that characterizes the usage of the remedies (in comparison to the ART which requires a rigid routine), pill burden of orthodox HIV medication, quest for alleviation of side effects of ARVs, and unbridled accessibility (26, 30) indeed compare with our findings. Additional motivators of the practices have been identified in these studies (17, 47, 48). Prior to our study, antidotal evidence did link the practice to periodic shortages of antiretroviral drugs, side effects associated with ARVs, and perceived efficacy of alternative medications. These were all confirmed by the current study.

While proponents of the use of alternative remedies argue that the practice empowers patients to treat themselves, and when used inappropriately, could pose significant risks. Some clinical studies show that herbal medicines might have the potential to alleviate symptoms, reduce viral load, and increase CD4+ cells for HIV-infected individuals and AIDS patients (33, 38). Of note, lower CD4+ counts of less than 350 cells/mm^3^ of blood is indicative of immune suppression and has been used as a clinical eligibility criterion for initiating ART (53). A normal CD4+ count ranges from 500 cells/mm^3^ to 1,500 cells/mm^3^ of blood. Any intervention or remedy that increases this count could improve the prognosis of HIV treatment. It must be noted, however, that aside increases in CD4+ cell count, a number of studies have associated the use of multiple medications with adverse consequences including treatment non-adherence (7, 45, 46). In addition to non-adherence to ARV regimen, it has been previously argued that the use of non-prescription remedies may potentiate low retention in ART clinics (26). The authors identified among others, reasons for low retention in ART care to include the need to travel long distances to receive medication, and preference for faith and religious remedies over orthodox treatment (26, 27). In a descriptive cross-sectional study conducted in the Upper West Region of Ghana, patients’ suboptimal adherence to ART therapy was mainly attributed to shortage of drugs (42%) and side effects of ARVs (17%) (4).

Other implications of non-prescription remedies’ use by HIV-positive person receiving ART include intrinsic toxicity of ingredients and drug–drug interactions. The studies by Liu et al. warn of liver toxicity and other adverse events from some herbal products as well as possible herb–drug interactions (29, 33, 38). One of the studies revealed that about 20% of HIV-patients used herbs that were very likely to compromise the effectiveness of ART (38).

The current study was interested in identifying background, socio-demographic, and clinical predictors of the use of non-prescription remedies. The multiple logistic regression model examined the practice of various potential predictors, and after adjusting for a number of covariates revealed that sex of ART client, religious affiliation, level of education, clinical parameters such as body mass index, and CD4+ cell count were all not significantly associated with the use of non-prescription remedies (Table 2).

The analysis confirmed the location of ART clinic as the only predictor of non-prescription remedies use. Thus, compared to clients from the Korle-Bu Fevers Unit, those from the Atua ART clinic were ninefold as likely to use non-prescription remedies (AOR = 8.84; 95% CI 2.829–33.720), and those from the St. Martin’s ART clinic, threefold as likely (AOR = 2.610; 95% CI 1.074–9.120). Based on the literature reviewed and discussed in this article, the arguments surrounding what determines the use of non-prescription remedies have focused on the flexibility that characterizes the usage of such remedies, pill burden of orthodox HIV medication, quest for alleviation of side effects of ARVs, and periodic shortages of antiretroviral drugs. While our study does not discount this, it adds a new line of conversation that the location of an ART clinic matters. Our collective experience supports this argument that location/environment matters—practices by service providers and service users in a cosmopolitan city versus district capital usually differ in many respects. Some of the clinic-specific factors that may explain why patients receiving ART in the two non-urban sites are more likely to use non-prescription drugs than those in other clinics include the caliber of health personnel. For example, the ART clinic at the Korle Bu Teaching Hospital is managed by a team of highly specialized physicians, pharmacists, and clinical psychologists. Second, the clientele of the clinic are likely to be better placed to reprimand and negative feedback for services given outside orthodox practices and treatment guidelines. This is in contrast to the other clinics run by nurses in the rural settings, whose patients are one large family and are more tolerant to certain behaviors.

Our study has a number of important limitations, which out to be acknowledged. The data presented in this article are derived from research conducted at four ART clinics in southern Ghana. Therefore, the limitation of applying the findings in other parts of the country is acknowledged. This is more so because HIV+ persons are not a homogenous group, hence the perspectives of those interviewed in this study might be different from what pertains elsewhere in Ghana. In this regard, large-scale quantitative and qualitative studies might be required to provide a more holistic picture about the use or misuse of non-prescription remedies by PLHIV in Ghana. Finally, the data were self-reported, and retrospective, making room for possible recall and other biases. These limitations notwithstanding, important lessons can be drawn from the findings in this article to inform policies and interventions that seek to promote access to ARV, adherence to ARVs, and retention in care.

## Conclusion

This study reveals the use of non-prescription remedies by PLHIV on ART to be a common practice. Usage is mostly self-initiated informed by perceived efficacy of the remedies. In a few instances, the remedies are used with the acquiescence of health workers. The site of ART clinic is a significant determinant of the use of non-prescription remedies. Patients and care providers at rural sites will need education about potential adverse effects of the use of non-prescription remedies concurrently with ART.

## Ethics Statement

This study was approved by the Ethical Review Committee of the Ghana Health Service, Research and Development Division, Accra, Ghana. Participation in the study conformed to the required ethical guidelines for the use of human subjects. The study proposal was reviewed and approved by the Ethical Review Committee of the Ghana Health Service, Research and Development Division, Accra (Protocol ID NO: GHS-ERC 03/11/13). Permission was granted from the facilities within which the study was conducted. Informed consent was obtained from all participants after the objectives and the methodology of the study was explained to them. Participation in the study was completely voluntary, and no financial or material benefits were given. The privacy and confidentiality of every participant were ensured throughout the study period. Identification numbers (and not names) were used to disguise identity. Every member of the data collection and analysis team was cautioned during the training sessions to maintain strict confidentiality and anonymity of study data and participants. The participants were all adults (18 years or older).

## Author Notes

Amos K. Laar has demonstrated research interests spanning HIV, maternal, infant, and young child nutrition. He has experience in planning, implementing, and disseminating community-based research. He has been a co-investigator of 10 successful research grants at the University of Ghana, and has published 32 articles in refereed journals. Awewura Kwara is an Infectious Diseases Specialist, with training in public health and tropical medicine. He is an Associate Professor of Medicine at Warren Alpert Medical School of Brown University. His research interest includes management HIV and TB coinfection. His research is in the area of TB and HIV treatment has made important contributions to international research efforts in the field of pharmacokinetics, pharmacogenetics, and drug–drug interactions between antiretrovirals and anti-TB drugs. Priscillia A. Nortey is a Lecturer at the Department of Epidemiology and Disease Control, School of Public Health, University of Ghana, Accra, Ghana. She is a Clinical Pharmacist worked most of her professional life in the public health delivery service of Ghana. In the past 10 years of her time in this service she was mostly involved in training of health personnel. She has been part of the teams involved in the development of several health management guidelines, notably guidelines for HIV and AIDS comprehensive care, pharmaceutical service, tuberculosis, and malaria and training health personnel in the provision of these services. She has also been a core member of clinical team for renal transplant and scoliosis surgery. Augustine K. Ankomah is an Associate Professor of Public Health at the Department of Population, Family and Reproductive Health, School of Public Health, University of Ghana, Legon, Accra. He has blend of academic, program implementation, research, and consultancy experience in HIV and reproductive health, with particular reference to most-at-risk populations. Over the past 20 years, he has conducted individual research, led international multi-center collaborative research and evaluation, published in peer-reviewed international journals, and presented at several international conferences. Michael P. K. Okyerefo is a Senior Lecturer, Department of Sociology, University of Ghana and a trained sociologist with varied research interests, including a focus on the nexus of religion and a host of socio-economic, political, and health processes in contemporary Ghana, Dr. Okyerefo’s research centers on two general areas of sociological inquiry, cultural sociology, and sociology of religion. He is a Principal Investigator in a research on Religious and Health Beliefs and Practices of Prayer Group members in Achimota Forest, Accra. Margaret Y. Lartey is a Professor of Medicine and has been working closely with HIV-infected patients in the area of clinical management. In Ghana, she is the most experienced HIV clinician managing the single largest clinic with 15,000 patients on roll and 6,000 on antiretrovirals. She contributes actively to drawing of guidelines and policies on the management of HIV infection and involved in the training of all cadres of health workers in HIV care as well as supervising and monitoring other sites.

## Author Contributions

AL is the principal investigator of the grant from which this manuscript metamorphosed. All the authors are co-investigators of the grant. AL conceived the study. AK and ML led the clinical components of the study design and tool development. PN and MO contributed to the development of study tool. ML supervised field work at the Korle-Bu Teaching Hospital. AL conducted the data analysis and drafted the first version of the manuscript. AA proofread the “Methods” section of the article. ML and AK contributed to the interpretation of the clinical data. All the authors read and approved the final version of the manuscript.

## Conflict of Interest Statement

The authors declare that the research was conducted in the absence of any commercial or financial relationships that could be construed as a potential conflict of interest. The reviewer, AA, and handling editor declared their shared affiliation, and the handling editor states that the process nevertheless met the standards of a fair and objective review.

## Abbreviations

AIDS, acquired immune deficiency syndrome; AOR, adjusted odds ratio; ARVs, antiretroviral; ART, antiretroviral therapy; CAM, complementary and alternative medicines; CSPro, Census and Survey Processing System; CL, confidence interval; GHP, Ghana Herbal Pharmacopoea; HIV, human immunodeficiency virus; UNAIDS, Joint United Nations program on HIV/AIDS; OTC, over-the-counter; PLHIV, persons living with human immunodeficiency virus; PMTCT, prevention of mother to child transmission; RAs, research assistants; SPSS, Statistical Package for the Social Science.
